# Maternal Postnatal Psychopathology Predicts Identity Diffusion in Young Adult Offspring

**DOI:** 10.3390/children12010024

**Published:** 2024-12-26

**Authors:** Jens Joas, Justine Hussong, Sena Aktürk, Kirstin Goth, Eva Möhler, Hannah Honecker-Gebauer

**Affiliations:** 1 Department of Child and Adolescent Psychiatry, Faculty of Medicine, Saarland University, 66421 Homburg, Germany; 2 Department of Child and Adolescent Psychiatry, Saarland University Hospital, 66421 Homburg, Germanykirstin.goth@uks.eu (K.G.);

**Keywords:** maternal psychopathology, identity diffusion, longitudinal study, criterion A, young adults

## Abstract

Background/Objectives: In the new conceptualization of personality disorders (PD) in ICD-11 and Diagnostic and Statistical Manual 5 Alternative Model of Personality Disorders (DSM-5 AMPD), identity development in terms of impaired personality functioning plays a central role in diagnostic guidelines and determining PD severity. On the one hand, there is a temporary identity crisis while keeping an integrated sense of identity and, on the other hand, there is pathological identity diffusion, which is associated with a high risk of a current or emerging PD. The latter is characteristic not only of borderline PD but of all personality disorders and should be detected as early as possible to prevent chronic illness and critical life courses. Maternal psychopathology is linked to several areas of child psychopathology (e.g., eating disorders, depression). In the current study, its potential to predict a child’s impaired identity development is investigated. Methods: A total of 101 mothers were asked about their health status 2 weeks after the birth of their child and when their child was 6 weeks, 4 months, 14 months and 5.5 years of age. Specifically, physical and psychological symptoms were assessed with SCL-90-R. In addition, their children were assessed in young adulthood regarding their identity development with the AIDA (Assessment of Identity Development in Adolescence) questionnaire. Linear regression models were used to investigate the amount of explanation of children’s identity diffusion by maternal symptom burden. Results: Maternal psychopathology significantly predicted identity diffusion at all time points with small effect sizes, while after 14 months, the explanation model showed a medium effect size. Conclusions: The present data suggest a relevant influence of maternal psychopathological symptoms on their children’s identity development in terms of functioning that has not yet been empirically shown in a longitudinal study. This finding highlights the importance of including further factors (particularly on the part of the child) in longitudinal studies and of investigating this clinically highly relevant relationship in greater depth.

## 1. Introduction

Since 2022, the new ICD-11 (International Classification of Diseases) [1] has been implemented step-by-step internationally, and the diagnostic guidelines for personality disorders (PDs) are changing fundamentally. The changes correspond to the recommendations in DSM-5 AMPD (Diagnostic and Statistical Manual 5 Alternative Model of Personality Disorders; research Section 3) for an alternative dimensional diagnostic of personality disorders [2]. Personality disorders are now viewed as a continuum of “no impairment” to “severe impairment” in basic domains of personality functioning. In both classification systems, ICD-11 and DSM-5 AMPD, the domain identity is included to denote an important area of functioning.

In DSM-5, the Level of Personality Functioning Scale (LPFS) separates the four functioning domains identity, Self-Direction, Empathy and Intimacy, where identity is described as the “experience of oneself as unique, with clear boundaries between self and others; stability of self-esteem and accuracy of self-appraisal; capacity for, and ability to regulate, a range of emotional experience”. In ICD-11, the functioning domains are conceptualized very similarly; identity is part of “aspects of self”, in contrast to aspects of interpersonal dysfunction, and impairment is described as problems with stability and coherence of identity or self-worth that may lead to severe disturbances in or even loss of sense of self.

The development of a stable identity is a central task of adolescence [3] without, of course, assuming that the formation and growth of identity is limited exclusively to adolescence. Erik H. Erikson paraphrased identity in 1968 [4] (p. 22) as follows: “identity formation employs a process of simultaneous reflection and observation, taking place on all levels of mental functioning, by which the individual judges himself in light of what he perceives to be the way in which others judge him in comparison to themselves and to a typology significant to them; while he judges their way of judging him in light of how he perceives himself in comparison to them and to types that have become relevant to him.” As early as 1956 [5] (p. 57), Erikson gave the following definition of identity: “a mutual relation in that it connotes both a persistent sameness within oneself (self-sameness) and a persistent sharing of some kind of essential character with others.” Based on Erikson’s fundamental theoretical insights, later theories have placed different emphases on the role of the individual and the role of society in the process of identity formation, for example. According to Ermann [6], identity is a connection in a temporary space between a particular individual and their society. Erikson’s identity theory has also been extended to include individual differences, problem-solving abilities, culture, social and further aspects (for a more in-depth comparison of Erikson and modern theories, see [7]). However, it is assumed normal for identity development to include identity crises, which usually pass after a while (compare Erikson [8] and his understanding of a phase-specific identity crisis). Identity diffusion, in contrast to concepts of developmental identity formation, is related to identity pathology and serves as a counterpart to identity integration. Kernberg, whose contribution essentially shaped the term personality disorder and also developed transference-focused psychotherapy (TFP) for the treatment of patients with borderline personality disorder, referred to identity diffusion as “the lack of an integrated self-concept and an integrated and stable concept of total objects in relationship with the self” [9] (p. 39). This would often lead to little or no commitment to jobs, goals and relationships, an avoidance of ambivalence and a painful sense of incoherence [10].

In recent decades, there has been a great deal of empirical evidence for the connection between aspects of identity and pathological symptoms. For example, there are correlations between identity diffusion and anxiety [11], troubled diffusion with depression and nonsuicidal self-injury (NSSI) [12], troubled diffusion and somatic symptoms [13], bulimia [14], substance abuse [15], delinquency [16] and personality disorders [17,18], especially borderline personality disorder [19,20,21,22]. In this interplay between identity and psychopathology, there is still a lack of findings on the underlying mechanisms and factors involved [23].

Based on the relationships between parents and offspring, correlations between trauma or maltreatment in childhood and identity diffusion [24] or the preposition of disorders (in personality disorders [25]) have been found. Furthermore, children of mothers with borderline personality disorder showed a disorganized attachment (e.g., rapid changes between attachment and avoidance, disorientation, freezing and fearful expressions) [26]. In general, maternal psychopathology is often linked to child psychopathology (here, in the case of depression, specifically also in negative affect and internalizing and externalizing factors [27]). In addition, Chiang and Bai [28] identified a bidirectional effect between internalizing and externalizing problems of the child and parental stress in a cross-lagged panel design.

To date, no studies exist describing maternal postnatal psychopathology as a predictor for young adult identity diffusion. In the current study, this relation will be investigated in a longitudinal setting over a time course of nearly 20 years.

## 2. Materials and Methods

### 2.1. Procedure

In this panel study, we asked mothers at different points in time about their subjective impairment due to physical and, in particular, psychological symptoms. At these time points, their children were 2 weeks, 6 weeks, 4 months, 14 months and 5.5 years of age. We also investigated the development of identity in their children aged 18/19 years.

### 2.2. Participants

Data were originally collected by Möhler et al. [29]. Our voluntary sample consisted of Caucasian mothers with one-child pregnancies. The inclusion criteria were term birth, APGAR scores greater than seven and infant weight above two and half kilograms. A generally good health was also necessary and tested over the first three postnatal examinations. The mothers were recruited via four large local maternity units, came from both urban and rural areas, needed to be able to speak and write German and were not allowed to have an acute psychiatric disorder or to take drugs (with the exception of a maximum 5 cigarettes a day, but no alcohol) or fetus-harming medication.

All participants read the participant information sheet and had the opportunity to ask questions. In addition, an informed consent form was read, signed and returned. Participation was voluntary and could be withdrawn at any time without giving any reasons. This study was conducted in accordance with the Declaration of Helsinki and approved by local ethics committees.

As can be seen in the participant flow (Table 1), 87 (86.14%) of the original 101 mothers still participated after 5.5 years. Of these 87 mothers, 51 children were successfully contacted at 18/19 years of age and assessed with the AIDA questionnaire. A total of 50 completed the AIDA questionnaire, leading to our sample size. As one mother failed to complete the SCL-90-R questionnaire when her child was 5.5 years old, the final sample size at this time was only 49.

### 2.3. Measures

Physical and psychological symptoms of mothers were assessed at all times with Symptom Checklist 90 revised (SCL-90-R; original [30]; German version [31]). SCL-90-R consists of 90 items, which are assigned to nine symptom scales (somatization, obsessiveness, insecurity in social contact, depressiveness, anxiety, aggressiveness/hostility, phobic anxiety, paranoid thinking, psychoticism) and finally provide three parameters for describing overall stress. The Global Severity Index (GSI) indicates the extent of impairment, in general, the Positive Symptom Distress Index (PSDI) measures intensity and the Positive Symptom Total (PST) quantifies the number of impaired symptoms. The procedure is considered to be valid in terms of face and content, even if its postulated factor and scale structure could not be consistently confirmed by factor analysis. According to Blanz [32] (p. 250), the reliability is excellent, with Cronbach’s alpha 0.97 for the GSI of the adult sample (the instrument is permitted for adolescents aged 14 and older). The values for the individual scales for the verification sample are not quite as good and vary between 0.75 and 0.87. The average time required to complete the questionnaire is given as 10–15 min [31].

The Assessment of Identity Development in Adolescence (AIDA) assesses identity development in terms of impairments in personality functioning in adolescents aged 12–18 years by self-report (±2 years). The test enables a dimensional differentiation between healthy and impaired identity development, which is assumed to be associated with a high risk of a current personality disorder, especially borderline PD [33]. The questionnaire contains 58 items with a 5-step answering format (0 = no to 4 = yes). All items are added up to obtain the total scale identity diffusion. For descriptive reasons, the total scale is divided into two domains of Discontinuity and incoherence (scales), each containing three different aspects of identity development (subscales). This reflects the theoretical origins and complexity of the concept and is supposed to facilitate a differentiated interpretation of the results and specific therapy planning. For a screening, it is sufficient to consider the total score. The items are coded towards pathology; thus, high scores suggest a high level of impairment. Scores clearly above the average (T-scores above 60 or above 70) denote plausible risk for a current or emerging personality disorder. The original version of AIDA was developed in the German language [34], and it shows good scale reliabilities with Cronbach’s alphas of 0.94 on the total level, 0.87 and 0.92 on the primary level and 0.69 to 0.84 on the subscale level. The total score “identity diffusion” shows high clinical validity with differing highly significant results and with a large effect size of d = 2.6 standard deviations between school samples and SCID-II diagnosed borderline PD patients.

### 2.4. Statistical Analysis

The data were analyzed with IBM SPSS Statistics, version 28. In order to investigate the influence of the mothers’ psychological stress on their children’s identity development, a linear regression with the GSI of the SCL-90-R was calculated for each of the 5 measurement points of the SCL-90-R as a predictor of the total score identity diffusion of the AIDA. Gauss–Markov assumptions were tested. Heteroscedasticity or autocorrelation did not occur at any time. A significance level of 0.05 was used for all statistical tests, and all results were rounded to 2 decimal places.

## 3. Results

### 3.1. Sample

When first included in this study, the mothers in our sample were between 26 and 45 years old. The vast majority (82%) of them were married; only one mother (2%) was divorced. Eight mothers (16%) were single. In terms of their level of education, they can be categorized as above average, as 82% (*n* = 41) of them had a high school diploma and more than half had a university degree (56%). The children were evenly distributed in terms of gender and weighed an average of 3531 g (SD = 400.18 g) at birth. They also had to fulfill the inclusion criteria mentioned above. All further details can be found in Table 2.

### 3.2. Descriptive Analysis

As can be seen in Table 3, the impairment due to physical and psychological symptoms is highest 2 weeks after birth (GSI *M* = 0.32, *SD* = 0.23), similar in the two subsequent periods and lowest after 5.5 years (GSI *M* = 0.18, *SD* = 0.20). Three mothers (6%) show a noticeable T-value at the beginning and one (2%) at each of the two latest measurement times. The range is greatest after 5.5 years (±1.2). On the AIDA overall identity diffusion scale, 13 participants (26%) have a value that can be interpreted as noticeably above average, i.e., indicating the presence of or a developing personality disorder (T > 60). In two (4%) cases of these, identity diffusion appears to be severely impaired as they have T-values above 70.

### 3.3. Results of Linear Regressions

The subjectively maternal psychopathology assessed via SCL-90-R at the time points 2 weeks (T1), 6 weeks (T2), 4 months (T3), 14 months (T4) and 5.5 years (T5) significantly predicted postpartum at time point T6 (child age 18/19 years) identity diffusion (assessed via AIDA) (T1: β = −0.30, t = −2.18, *p* = 0.03; T2: β = −0.30, t = −2.16, *p* = 0.04, T3: β = −0.30, t = −2.19, *p* = 0.03; T4: β = −0.40, t = −3.05, *p* < 0.01; T5: β = −0.28, t = −2.02, *p* = 0.05 [0.049]). In addition, maternal psychopathology at all time points explained a significant proportion of variance in identity diffusion (T1: adjusted R^2^ = 0.07, F(1,48) = 4.74, *p* = 0.03; T2: adjusted R^2^ = 0.07, F(1,48) = 4.65, *p* = 0.04; T3: adjusted R^2^ = 0.07, F(1,48) = 4.79, *p* = 0.03; T4: adjusted R^2^ = 0.15, F(1,48) = 9.29, *p* < 0.01; T5: adjusted R^2^ = 0.06, F(1,47) = 4.10, *p* = 0.05 [0.049]). According to Cohen [35] (p. 412), T1, T2, T3 and T5 are small effects and T4 is a medium effect. The results are also shown in Table 4.

## 4. Discussion

In our study, we investigated the potential of maternal psychopathology at different time points to predict identity diffusion in their children at 18/19 years of age, starting shortly after birth. There were small effects at four of the time points and a medium effect 14 months after birth. In light of our data, we presume the persistence of maternal postnatal depressive symptoms until 14 months postnatal age makes children specifically vulnerable. According to data from the US National Comorbidity Survey, identity diffusion explains the variance in psychopathology generally [36]. Identity diffusion appears to be associated with a range of psychopathologies, not only borderline personality disorder with chronic emptiness, chronic boredom and affective dysregulation [37]. Impaired identity functioning is officially a diagnostic criterion in the Alternative Model of Personality Disorders (AMPD; research Section 3) in the DSM-5 [2]. In ICD-11 (for a summary of changes in DSM-V and ICD-11, see [38]), radical changes according to the guidelines for diagnosing PD are implemented [39]. Instead of following the familiar typification, the focus is now on general impairments of self- and interpersonal functioning and their classification according to a degree of severity, ranging from mild to severe. Similar to the DSM-5 AMPD, impairment of personality functioning is a central component to evaluate PD severity, while identity diffusion is included under the functioning aspects of self. Existing theories and empirical studies have linked identity diffusion, in particular, to borderline personality disorder [40,41,42]. High levels of identity diffusion have also been associated with less favorable treatment outcomes in borderline personality disorder [43].

A longitudinal analysis of interviews [44] showed the consequences of identity diffusion. Participants aged between 25 and 29 showed qualitatively reduced or haphazard activity in response to changing living conditions. They perceived only a few new elements of meaning-making and occasionally gave up their life orientation. In other studies, unresolved attachment was associated with identity diffusion [45], and individuals with identity diffusion showed less secure attachment [46]. Identity diffusion seems also to imply low autonomy, self-esteem and identity [47]. According to Hamer and Bruch, shyness was positively associated with identity diffusion in adolescence [48]. With regard to the five-factor model of personality, there was a positive correlation between identity diffusion and neuroticism and a negative correlation with agreeableness [49]. Further findings show links between identity diffusion and grandiose self-expression [50] and that individuals with identity diffusion tend to have problems adapting to the university context [51]. It, therefore, seems important to detect identity diffusion at an early stage in order to be able to act. If identity diffusion was diagnosed at an early stage, it may be treated in adolescence with quite effective manualized therapies [52].

Maternal psychopathology has been linked to a variety of child psychopathologies (e.g., eating disorders [53], depression [54], anxiety [55]). If, according to our results, maternal psychopathology can also be regarded as a possible risk factor for long-term identity diffusion in their offspring, then this risk factor must be taken into consideration in preventive efforts, and potential personality dysfunction could be screened for in childhood. Screening instruments already exist as parental assessment in childhood (e.g., LoPF-Q 6-18 PR [56]). However, screening and treatment of identity diffusion appear to be appropriate not only in childhood but even in the first years of study, as they can have a positive effect on academic performance [57], for example.

The mother–child interaction in the first years after birth could play a crucial role in explaining the specific correlation found in our study. In fact, several studies have shown that the mother–child interaction is significantly altered in the context of maternal psychopathology (e.g., [58]). Furthermore, the mother–child interaction has been demonstrated to be vital for children’s longitudinal development by multiple studies, e.g., the Finnish birth cohort examination [59].

### 4.1. Limitations and Future Research

Identity development is a complex and individual process in which a person has an influence on their environment but is also influenced by it. Regarding the cause of our solid and repeated correlation between maternal postnatal psychopathology and young adult identity diffusion, a variety of factors play a role in identity development; however, these have not all been covered by this study. Culture, for example, provides a context that can give individual opportunities but also restrictions. It can define desirable values, ideas and even behavior. Family relationships, social support and stress are markers of experienced well-being, including psychosocial well-being. As identity development can be subsumed under psychosocial development, the influence of family and social groups cannot be ruled out. On the part of the individual, identity can be impaired by early traumatic experiences (e.g., emotional or physical abuse) by destabilizing, questioning and re-evaluating existing identity commitments. Biological factors, whether inherited from birth or markers like puberty, growth and maturation, are directly or indirectly related to later self-image, self-esteem and perception of the environment. Due to the specific nature of the present sample, and in order to improve the generalizability of our results, more studies, including a clinical sample, are required to verify the validity of the relationship. According to the current research, adolescents with mental illness are more likely to have impaired identity development. Furthermore, determining whether maternal psychopathology also has an impact on adolescents in clinical care and to what extent would be of interest. Moreover, in our study, only the mother’s judgment of her own psychopathology was used in self-reporting. Finally, it should be noted that both the SCL-90-R and AIDA have the same informative value as screening and that professional judgment is required to determine pathology. Nevertheless, the extent of influence of maternal psychopathology appears to be considerable.

### 4.2. Clinical Relevance

This study is the first to empirically demonstrate a relation between maternal psychopathology in the first postnatal year and identity diffusion in the offspring over an almost 20-year longitudinal course of assessment. If the basis of this relationship is—in part—the mother–child interaction, these results not only underline that personality functioning might be formed in very early childhood. Specifically, identity development in early childhood is a complex process that is significantly influenced by a child’s experiences, activities and relationships with others. It also emphasizes the necessity of early screenings for young mothers, especially because nearly none of the mothers in our sample seem to be clinically ill. These findings show that the overall well-being of mothers in the first year after birth should be carefully monitored and supported.

## Figures and Tables

**Table 1:** Participant flow.

	N	%
N at child’s age of 2 weeks (T1), 6 weeks (T2), 4 months (T3)	101	100
n at child’s age of 14 months (T4)	98	97.03
n at child’s age of 5.5 years of life (T5)	87	86.14
Reached children of legal age	51	50.50
Final sample (i.e., completed questionnaires of the mothers and their children were available) (one exception, T5 had 49, which corresponds to 48.51%)	50	49.50

**Table 2:** Sample description (*n* = 50) at T1 (T1 = 2 weeks postpartum).

Characteristic	*M*	*SD*	Min.	Max.
Age of mother in years	33.88	4.02	26	45
Child’s birth weight in grams	3531.00	400.18	2770	4500
**Marital status**	**N**	**%**		
- Single.	8	16		
- Married.	41	82		
- Divorced.	1	2		
**Child’s gender**	**N**	**%**		
- Female.	26	52		
- Male.	24	48		
**Highest school-leaving certificate of mother**	**N**	**%**		
- Secondary school.	9	18		
- Grammar school.	13	26		
- University of applied science/University.	28	56		

**Table 3:** Descriptive statistics of SCL-90-R and AIDA (N = 50, at T5 *n* = 49).

SCL-90-R: GSI	*M*	*SD*	Min.	Max.
2 weeks postpartum (T1)	0.32/T = 50.06	0.23/T = 6.32	0.04/T = 37	1.08/T = 63
n T > 60	3 (6%)			
n T > 70	0 (0%)			
After 6 weeks (T2)	0.22/T = 46.74	0.15/T = 6.19	0.01/T = 32	0.67/T = 59
n T > 60	0 (0%)			
n T > 70	0 (0%)			
After 4 months (T3)	0.21/T = 45.58	0.17/T = 7.76	0.00/T = 27	0.72/T = 60
n T > 60	0 (0%)			
n T > 70	0 (0%)			
After 14 months (T4)	0.24/T = 46.36	0.21/T = 8.50	0.00/T = 27	1.04/T = 64
n T > 60	1 (2%)			
n T > 70	0 (0%)			
After 5.5 years (T5)	0.18/T = 43.67	0.20/T = 7.77	0.00/T = 27	1.20/T = 65
n T > 60	1 (2%)			
n T > 70	0 (0%)			
**AIDA: total score identity diffusion** (T6)	150.12/T = 54.10	25.58/T = 8.30	85/T = 38	200/T = 75
n T > 60	13 (26%)			
n T > 70	2 (4%)			

**Table 4:** Results of the computed simple linear regressions of each survey time point (T1: 2 weeks postpartum, T2: 6 weeks, T3: 4 months, T4: 14 months, T5: 5.5 years) (N = 50, at T5 *n* = 49). T6 children were interviewed at the age of 18/19. B represents unstandardized regression weights; SE B is the standard error for B. Beta indicates the standard regression weights. F2 was calculated with adjusted R^2^. * *p* < 0.05, ** *p* < 0.01, ’ *p* = 0.049.

Criterion	Predictor	*B*	*SE B*	*β*	*T*	*p*	Fit/*R*^2^	Adjusted *R*^2^	*F*	*p*	*f* ^2^
**AIDA: total score identity diffusion at T6**							0.09	0.07	4.74	0.03 *	0.08
	SCL-90-R GSI at T1	−33.13	15.22	−0.30	−2.18	0.03 *					
							0.09	0.07	4.65	0.04 *	0.07
	SCL-90-R GSI at T2	−51.25	23.76	−0.30	−2.16	0.04 *					
							0.09	0.07	4.79	0.03 *	0.08
	SCL-90-R GSI at T3	−46.63	21.31	−0.30	−2.19	0.03 *					
							0.16	0.15	9.29	<0.01 **	0.17
	SCL-90-R GSI at T4	−48.59	15.94	−0.40	−3.05	<0.01 **					
							0.08	0.06	4.10	0.05 *’	0.06
	SCL-90-R GSI at T5	−37.15	18.36	−0.28	−2.02	0.05 *’					

## Data Availability

The raw data supporting the conclusions of this article will be made available by the authors without undue reservation.
